# Effect of yeast culture feed supply on growth, ruminal pH, and digestibility of fattening calves

**DOI:** 10.1002/fsn3.2238

**Published:** 2021-03-22

**Authors:** Omar Maamouri, Mondher Ben Salem

**Affiliations:** ^1^ National Institute of Agronomic Research of Tunisia University of Carthage Manzah Tunisia

**Keywords:** calves, growth, ruminal pH, yeast culture

## Abstract

This study was carried out to predict the effects of yeast culture on growth, intake, and digestibility in vitro of calves for fattening. The trial involved 16 fattening calves divided into two homogeneous groups (*n* = 8), based on the initial body weight (414 ± 25.1 kg and 416 ± 24.4 kg) (*p* = .96) for the control group (C) and the experimental group (Y). The ration is wheat straw and concentrate. Group Y additionally receives a quantity of 10 g/calf/day of yeast culture. The quantity of feed was 5 kg DM/calf/day of wheat straw and 8kg DM/calf/day of concentrate. This trial lasted 112 days. We measure the weights every two weeks with a cattle scale and also the rejected quantities of wheat straw at each measure. A significant increase (*p* < .01) in total average daily gain (adgT) during the trial was observed at 200g/calf. In addition, an increase (*p* < .01) in the final weight gain (FWG) was observed at 19 kg/calf for group Y compared with group C. Intake does not vary with the yeast culture. The feed conversion rate (FCR) was lower for group Y compared with group C (7.8 ± 0.2 versus 9.6 ± 0.5, *p* < .01). We observed a notable increase in ruminal pH for group Y compared with group C.

## Graphical abstract

This study was carried out to predict the effects of yeast culture on growth, intake, and digestibility in vitro of calves for fattening. Improved performance has been shown following supplementation in yeast culture.

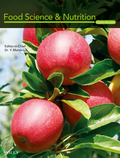

## 1 INTRODUCTION

The cattle breeding in Tunisia is characterized by the use of the Holstein breed, which has a high production potential. Fodder resources are often insufficient and of average nutritional quality. The farmer is forced to bring large quantities of concentrated feed, in particular the fattening activity, hence the risk of acidosis, particularly subclinical. *Saccharomyces cerevisiae* have been used as preventer supplement against diarrhea and other digestive system problems in livestock (Chaucheyras‐Durand et al., 2008). They also give production benefits, reduced digestive problems, and better health of animals in cost‐effective manners (Huber, 1997). Dietary supplementation of yeast culture has a positive effect on feed intake, which ultimately enhances ruminant growth (Robinson and Erasmus, 2009) and production efficiency (Poppy et al., 2012). Then, adding yeast culture to ruminant diets with large amounts of readily fermentable carbohydrates may reduce lactate concentration, improve rumen pH, and boost performance. Also, the benefits from adding a yeast culture to the diet may be heightened in diets with a large amount of starch.

This acidosis often results in reduced ingestion and production (growth). Beauchemin et al., (2003) reported that the addition of the yeast *Saccharomyces cerevisiae* stimulates the development of lactate‐consuming bacteria in the rumen, which could result in improved ruminal conditions and improved performance. To enhance their yields and increase their profits, these producers increase the percentages of concentrate in animal feed, without taking into account the risks of metabolic diseases such as acidosis induced by this abuse. Contributing to a decrease in production, the fall in ruminal pH below the edge of 5.6 induces this disorder. To avoid this risk, several surveys have shown that feed additives seek to eliminate latent acidosis in ruminants, in particular, studies on the yeast *Saccharomyces cerevisiae* (Chaucheyras‐Durand & Durand, 2010; Chaucheyras‐Durand et al., 2008; Desnoyers et al., 2006). They make it possible to maintain good animal health following digestive comfort, and this improved their production. Although the yeast cell is around 4 µm, many consider it to be among the oldest of human industrial microorganisms. The yeast cell is used for the fermentation and breaking down of carbohydrates into useful products such as CO_2_ and ethanol, and it can also provide beneficial health benefits through new biotechnology in its production process. Yeast cells break down carbohydrates into simple sugars such as glucose, which is taken up by the yeast cell to be used as an energy source for reproduction and other metabolic processes. Multitudes of by‐products such as carbon dioxide, alcohols, organic acids, peptides, amino acids, and refined functional carbohydrates and many other nutrients are processed into a finished product called yeast, which is used as a feed additive in the food industry and as an additive animal feed. The objective of this research was therefore to determine the effect of the contribution of the yeast culture *Saccharomyces cerevisiae* on fattening calves, where the ration is relatively rich in concentrate.

## 2 MATERIALS AND METHODS

The experimental procedures were approved by the Committee of Animal Experiments (CEEA), Tunisia.

### 2.1 Animals and management

The trial took place in northeastern Tunisia for 112 days on 16 Holstein calves, which were divided into two homogeneous groups (eight calves per group) according to age (15 months) and body weight 415 ± 16.9 kg, and received the same ration composed of wheat straw and concentrate feed. Each calf in the yeast group Y also received 10 g/calf/day of powder of the *Saccharomyces cerevisiae* yeast culture with the concentrate. The ration includes wheat straw 5 kg DM/calf/day and 8 kg DM concentrate for the control group C. The yeast culture used is the yeast *Saccharomyces cerevisiae* cultivated on a sucrose and cane molasses media and dried with processed cereal by‐products. It is designed to be administered to all classes of livestock where yeast is desired. Its chemical composition is shown in Table 1.

**TABLE 1:** Chemical composition and nutritive value of feeds

	Concentrate	Wheat straw	Yeast culture
DM (g/kg)	900	920	900
DM composition (g/kg DM)			
CP	170	48	230
CF	60	330	10
Ash	40	75	30
OM	950	925	970
FM	35	nd	2
ME (MJ/kg) DM)	7.6	3.2	nd
PDIE (g/Kg DM)	116	51.9	nd
PDIN (g/Kg DM)	117	33	nd

Note

Abbreviations: and PDIN, when nitrogen is the limiting factor for rumen microbial activity; CP, crude protein; DM, dry matter; FM, fat matter; ME, metabolized energy (MJ/kg). The nutritional values for nitrogen are expressed as digestible protein in the intestine or PDI (in g/kg); nd, not determinate; OM: organic matter; PDIE, when energy is the limiting factor for rumen microbial activity.

### 2.2 Experimental design

We measured the weights every two weeks with a cattle scale. We also calculated average daily gains (adg), average daily gain mean (adgm) and final weight gain (FWG), and feed conversion rate (FCR). The rejected quantities of wheat straw are also weighed at each check with a scale. It should be noted that the full amount of concentrate was ingested.

### 2.3 Sampling and chemical analysis

We established the chemical composition of the different feed resources in the animal nutrition laboratory at the National Institute of Agronomic Research in Tunisia. We determine the nutritive contents of experimental aliments following the method described by Sauvant (1981). We dried the samples of diets in a forced‐air oven at 105°C for 24 hr to determine DM. Dried samples were then ground through a 1‐mm screen. The Kjeldahl method (AOAC, 1984) determined the crude protein.

We performed the calculations of energy values using the approach and the equations proposed by INRA (INRA, 1978, 1988). We express the nutritional values for nitrogen as digestible protein in the intestine or PDI (in g/kg). The PDI values are indicated (INRA, 1978, 1988): PDIE, when energy is the limiting factor for rumen microbial activity; and PDIN, when nitrogen is the limiting factor for rumen microbial activity.

We conducted rumen liquid out. We did this act five at six hours after distribution of concentrate (Sauvant et al., 1999). We measure the samples with a pH meter. We had these measures out during the weeks of weighting control measures.

### 2.4 Statistical analysis

The results of the effects of diets on the measured parameters (growth parameters, feed intake, feed conversion rate, and ruminal fluid pH) were subjected to analysis of variance with the GLM procedure of the statistical package SAS User’s Guide (2000) and compared by *t*‐test diff.

The statistical model was as follows: Yij = μ + Ri + eij.

With: Yij: measured parameter.

μ: overall mean.

Ri: fixed effect of diet (i = 1, 2).

eij: residual error term.

Significance was declared at *p* < .05 unless otherwise declared.

### 2.5 In vitro fermentation parameters

We performed a determination of the total gas on the contents of the rumen filtered from the calf. In syringes, we set 0.3 g of a substrate (concentrate ground to 1 mm), 10 ml of rumen juice filtrated from ruminal fluid, and 20 ml of artificial saliva. Then, we put the syringes vertically in a water bath at 39°C; we look at the reading every 2 hr after mixing syringes until a bearing (Orskov and Macdonald, 1979).

## 3 RESULTS

### 3.1 Chemical composition of food

The chemical composition of the feed is presented in Table 1. For wheat straw, it has a low content of crude protein (CP) (48 g/kg DM) and metabolized energy (ME) (3.2 MJ/kg DM). We could consider that the CP content is deficient (Norton, 1994). For concentrate feed, the CP and ME contents are 170 g/kg DM and 7.6 MJ/kg DM. Yeast culture has a high nutritional value that allows a high crude protein content with 230 g/kg DM and a low crude fiber content value 10 g/kg DM.

### 3.2 Growth performance

The results showed a notable increase (*p* < .01) in the final weight gain (FWG) of 19 kg/calf for group Y compared with group C (Table 2). Likewise, we illustrate that the addition of 10 g of *Saccharomyces cerevisiae* yeast culture/calf/day increases the average daily gain mean (adgm), from 1,230 g/calf/day to 1,430 g/calf/day (*p* < .01) (Table 2).

**TABLE 2:** Effect of yeast culture on weight and average daily gain

	Group		*SEM*	*p*‐value
	C	Y		
Pretrial weight, W0 (kg)	414 ± 25.1	416 ± 24.4	70.1	.96
Final trial weight, W7 (kg)	535 ± 25.1	556 ± 23.3	68.5	.55
FWG (kg)	121^a,b^ ± 5.5	140^a,b^ ± 3.8	13.4	.01
adg (mean) (kg/calf/day)	1.23^a,b^ ± 0.06	1.43^a,b^ ± 0.03	0.14	.01

^a,b^ Mean values with different letters in the same row are significantly different; *SEM*, standard error mean; (±), standard deviation; W, weight (kg); adg (mean), average daily gain mean measured during the trial (kg/calf/day); FWG, final weight gain; C, control group; Y, culture yeast group.

### 3.3 Feed intake and feed conversion rate

Based on the results found, we did not report any significant differences between the two groups in the feed intake throughout the trial. The amount of the average daily total intake is 10.738 kg/calf/d against 10.736 kg/calf/d, and the total amount ingested during the whole trial is 75.2 kg/calf/trial period against 75.1 kg/calf/trial period, respectively, for group C and group Y. The feed intake did not differ between animals of two groups throughout the trial. Statistical analysis also revealed that there was no significant distinction (*p* > .05) in the feed conversion ratio between the two groups except for the mean conversion rate (mean FCR) with 9.6 versus. 7.8, respectively, for C and Y groups (*p* < .01) (Table 3).

**TABLE 3:** Effect of yeast culture on intake and feed conversion rate

	Group		*SEM*	*p*‐value
	C	Y		
Average intake (kg DM/calf/day)	10.73 ± 0.009	10.74 ± 0.009	0.03	.85
Total intake (kg DM/calf/trial)	75.2 ± 0.06	75.1 ± 0.06	0.2	.70
Feed conversion rate	9.6^a,b^ ± 0.5	7.8^a,b^ ± 0.2	1.2	.01

^a,b^ Mean values with different letters in the same row are significantly different; *SEM*, standard error mean; (±), standard deviation; C, control group; Y, culture yeast group.

### 3.4 In vitro fermentation parameters

The addition of the *Saccharomyces cerevisiae* yeast culture did not modify the fermentation parameters (*p* > .05) (Table 4). Gas production in vitro in 100‐mL glass syringes undergoes rapid development after incubation. After 24 hr of incubation, diet C records the lowest significant amount (*p* < .05) of gas 64 ml / 0.3 g of DM and is followed by the diet added by the yeast culture, which yields the largest amount 68.5 ml/0.3 g. We report the kinetic parameters of in vitro fermentation of different substrates, deduced from the exponential model of Orskov and Macdonald (1979) in Table 4. The food of group Y is the most (*p* < .0001) fermented by the ruminal microbiota (0.03 (h^‐1^)) followed by the food of C group (0.02 (h^‐1^)). In vitro fermentation of two substrates depends on a lag phase, evidenced by the negative value of the soluble fraction (a) −0.6 ml/0.3 g DM and 1.8 ml/0.3 g DM for C and Y diets, which explains its low degradation. This lag phase appears to be due to the time required for microorganisms to adhere to and colonize dietary fibers. Regarding the other parameters, the predicted values suggest that the organic matter digestibility (OMD) of group C is 77.7% and 81.7% for Y one with a considerable difference (*p* < .05). The same is true for the ME released by the different substrates (*p* < .05), 11.4 MJ versus. 12.1 MJ for the substrates of food groups C and Y. And the volatile fatty acids (VFA) recorded the different values were 1.47 mmol/syringe for group food C against 8.95 mmol/syringe for group food Y with a notable difference (*p* < .05) (Table 4).

**TABLE 4:** Parameters a, b, c, and a + b of the nonlinear model of gas production and the parameters estimated from the gas produced at 24 hr: comparison of the two diets C and Y

	C	Y	*SEM*	*p*‐value
a (mL)	−0.6^a,b^ ± 1.6	1.8^a,b^ ± 1.02	0.4.10^–4^	<.0001
b (mL)	140.4^a,b^ ± 22.4	118.1^a,b^ ± 6.2	0.36.10^–5^	<.0001
c (h^−1^)	0.02^a,b^ ± 0.006	0.032^a,b^ ± 0.003	0	<.0001
a + b (mL)	139.8^a,b^ ± 24	116.3^a,b^ ± 7.2	‐	<.0001
Prod gas 24 hr (mL)	64^a,b^ ± 1.4	68.5^a,b^ ± 0.7	1.11	.05
OMD (%)	77.7^a,b^ ± 1.2	81.7^a,b^ ± 0.6	0.99	.05
EM (MJ)	11.38^a,b^ ± 0.22	12.08^a,b^ ± 0.1	0.17	.05
VFA (mmol/syringe)	1.47 ± 0.02	8.95 ± 10.4	7.36	.05
EM (Kcal)	2,719.4^a,b^ ± 53	2888^a,b^ ± 26.5	41.9	.05

Note

a: quantity of gas produced (mL) immediately from the substrate

b: gas production potential.

c: gas production speed.

^a,b^ Mean values with different letters in the same row are significantly different; *SEM*: standard error mean; (±): standard deviation; C: control group; Y:culture yeast group; OMD: organic matter digestibility; ME: metabolized energy; VFA: volatile fatty acid.

### 3.5 Ruminal pH

The respective ruminal pH values for the two groups are about 6 or less, the smallest value is 5.80. The results show a notable variation in ruminal pH between the two groups in the middle and at the end of the test period. We note a significant increase in ruminal pH for group Y compared with group C at the 4th, 5th, 6th, and 7th controls, as well as for the ruminal pH mean, which may be due to the culture yeast supply (Table 5).

**TABLE 5:** The effect of yeast culture on the ruminal pH

	C	Y	*SEM*	*p*‐value
pH1	5.80 ± 0.02	5.80 ± 0.04	0.03	<.9
*pH2*	5.89 ± 0.02	5.90 ± 0.04	0.03	<.6
*pH3*	5.90 ± 0.02	5.93 ± 0.03	0.03	<.4
*pH4*	5.93^a,b^ ± 0.02	5.96^a,b^ ± 0.02	0.03	.05
*pH5*	5.93^a,b^ ± 0.03	5.96^a,b^ ± 0.04	0.04	.03
*pH6*	5.92^a,b^ ± 0.02	5.97^a,b^ ± 0.02	0.03	.008
*pH7*	5.90^a,b^ ± 0.05	5.98^a,b^ ± 0.05	0.04	.01
*Average pH*	5.90^a,b^ ± 0.05	6.00^a,b^ ± 0.04	0.04	.003

^a,b^ Mean values with different letters in the same row are significantly different; *SEM*, standard error mean; (±), standard deviation; C, control group; Y, culture yeast group.

## 4 DISCUSSION

The precise mode of action by which the yeast culture, which is mainly derived from *Saccharomyces cerevisiae*, improves the performance of cattle has attracted the attention of several researchers around the world. It is clear from these research efforts that yeast culture supplements can beneficially modify microbial activities, fermentation, and digestive functions in the rumen. Research has shown that viable yeast culture preparations can stimulate specific groups of beneficial bacteria in the rumen, and provide mechanisms that may explain their effects on animal performance. The experiments conducted by El’ Hassan et al. (1993) and Hancock et al. (1994) on young calves revealed a significant increase in average daily gain (adg) when the animals were on an acidogenic diet, and this could be due to the effect of yeast, which reduces the fermentative disorders in the rumen caused by the concentrate feed (Desnoyers, 2008). An improvement in growth rate was obtained with a yeast *Saccharomyces cerevisiae* administered to growing dairy heifers (Ghazanfar et al., 2015).

Regarding ingestion, our results are consistent with those of Desnoyers et al., (2006), who noticed that the amount consumed does not vary with the yeast supplement in the diet. The lack of distinction on ingestion can be explained by the fact that the straw is not distributed at will. We suggest this proposal because another study led by Majdoub‐Mathlouthi et al., (2011) found that the addition of yeast increases the voluntary consumption of forage. Mutsvangwa et al., (1992) presented that the inclusion of yeast in an acidogenic diet leads to an increase in dry matter intake in beef herds. Other work directed by Moncoulon and Auclair (2001) showed a considerable decrease of 2.6% in the quantity of dry matter ingested. This trend can be interpreted by the fact that the yeast effect on intake is weak with a diet rich in concentrate feed (high energy intake) due to the metabolic satiety already established following the considerable production of VFA from carbohydrates and acetate and propionate from lactic acid. Thus, intake may increase with a diet rich in fiber following the direct action of the yeast on the communities, which degrade the fiber within the rumen through its action on the level of oxygen consumption (Marden et al., 2008) and increase the fibrolytic activity by accelerating digestive transit and increasing dry matter intake (Chaucheyras‐Durand & Durand, 2010). The effects of yeast culture on animal productivity depend on the strain. Thus, not all yeast culture preparations are equivalents to ineffectiveness. One possible explanation for this effect is that a low intake of dry matter does not provide the microbial population with sufficient soluble growth factors. Like organic acids, B vitamins, and amino acids, Callaway and Martin (1997) suggested that the yeast culture provides soluble growth factors, which stimulate the growth of cellulolytic bacteria and the digestion of cellulose. However, the mechanism for improving the dry matter ingested with supplementation in yeast culture has not been clearly defined. It is clear from research efforts that yeast culture supplements can advantageously modify microbial activities, fermentation, and digestive functions in the rumen (Denev et al., 2007). Yeast cultures are very beneficial in the rumen. Several reasons for improving ruminal fermentation from the feed of the yeast culture have been suggested. Numerous studies (Alshaikh et al., 2002; Lila et al., 2004; Tricarico et al., 2006; Chevaux and Fabre, 2007) have documented the positive effects of yeast culture not only on the rumen environment but also on improving microbial activities.

Yeast culture supply influences the metabolism of ruminal lactic acid. Yeast culture prevents the buildup of excess lactic acid in the rumen when cattle are fed feed containing highly fermentable carbohydrates. Sullivan and Martin (1999) reported that adding a culture of the yeast *Saccharomyces cerevisiae* to the diet of dairy cows improved lactate utilization and cellulose digestion. While the herd is in latent acidosis, it is imperative to get involved in sustaining the pH to an average between 6 and 6.4 which we consider to be the optimal range for rumen fermentation, fiber digestion, and offering to high‐yielding animals an adequate state of health (Sauvant et al., 1999). Acidosis results from an almost permanent imbalance between a high production of volatile fatty acids and a low yield of the salivary buffer, when the ration is unsuitable, with a concentrate and a very limited forage, which reduces the production of saliva to buffering power, and in the event of a poor diet and poor dietary transition (Maillard, 2008). Thus, this ration used seems to be acidogenic and crucial to act in the regulation of the ration and to eliminate the risk of acidosis. Below these values, we consider the herd in a state of acidosis.

In conclusion, this trial is used to study the interest of yeast culture as a feed additive to modulate rumen microbial fermentation and improve the production of fattening calves. The results show that the addition can improve animal production. There was a considerable increase in adgm and final weight gain (FWG). There was an improvement in the mean feed conversion ratio in favor of the animals that received the yeast culture. The contribution of the *Saccharomyces cerevisiae* yeast culture improved the fermentation parameters (OMD, VFA, and ME concentration). Thus, a noticeable increase was observed in ruminal pH with the addition of the yeast culture in the concentrate.

## 5 ANIMAL WELFARE STATEMENT

The authors confirm that they have followed the Committee of Animal Experiments (CEEA) of Tunisia for the protection of animals used for scientific purposes.

## CONFLICT OF INTERESTS

The authors declare that they have no conflict of interest.
